# Prevalence of G6PD deficiency and distribution of its genetic variants among malaria-suspected patients visiting Metehara health centre, Eastern Ethiopia

**DOI:** 10.1186/s12936-022-04269-5

**Published:** 2022-09-08

**Authors:** Tassew Tefera Shenkutie, Desalegn Nega, Asrat Hailu, Daniel Kepple, Logan Witherspoon, Eugenia Lo, Meshesha Tsigie Negash, Aderaw Adamu, Shewayiref Geremew Gebremichael, Bokretsion Gidey, Geremew Tasew, Sindew M. Feleke, Tadesse Kebede

**Affiliations:** 1 Department of Medical Laboratory Sciences, Debre Berhan University, Debre Berhan, Ethiopia; 2 Department of Microbiology, Immunology and Parasitology, Addis Ababa University, Addis Ababa, Ethiopia; 3 Bacterial, Parasitic, and Zoonotic Diseases Research Directorate, Ethiopian Public Health Institute, Addis Ababa, Ethiopia; 4 National Data Management Centre for Health, Ethiopian Public Health Institute, Addis Ababa, Ethiopia; 5 Department of Biological Sciences, University of North Carolina, Charlotte, NC USA; 6 School of Data Science, University of North Carolina at Charlotte, Charlotte, NC USA; 7 Department of Medical Laboratory Sciences, Wollo University, Dessie, Ethiopia

**Keywords:** Glucose-6-phosphate dehydrogenase, *Plasmodium vivax*, *Plasmodium falciparum*, Primaquine, Metehara, Ethiopia

## Abstract

**Background:**

Glucose-6-phosphate dehydrogenase (G6PD) is cytosolic enzyme, which has a vital role for the integrity and functioning of red blood cells. Lower activity of this enzyme leads to the occurrence of acute haemolytic anaemia after exposure to oxidative stressors like primaquine. Primaquine is an important drug for the radical cure of *Plasmodium vivax* and blocking transmission of *Plasmodium falciparum*, and thereby enhancing malaria elimination. However, there is a need to identify G6PD deficient individuals and administer the drug with caution due to its haemolytic side effects. The main objective of this study is to determine the prevalence of G6PD deficiency among malaria-suspected individuals.

**Methods:**

A facility-based cross-sectional study was conducted from September 2020 to September 2021 in Metehara Health Centre, Eastern Ethiopia. A structured questionnaire was used to collect the socio-demographic and clinical information of the study participants. Capillary and venous blood samples were collected based on standard procedures for onsite screening, dried blood spot preparation, and malaria microscopy. The G6PD enzyme activity was measured by careSTART™ G6PD biosensor analyzer. Data was entered and analysed by SPSS.

**Results:**

A total of 498 study participants were included in the study, of which 62% (309) were males. The overall prevalence of G6PD deficiency based on the biosensor screening was 3.6% (18/498), of which 2.9% and 4.8% were males and females, respectively. Eleven of the G6PD deficient samples had mutations confirmed by G6PD gene sequencing analysis. Mutations were detected in G267 + 119C/T, A376T, and ChrX:154535443. A significant association was found in sex and history of previous malaria infection with G6PD deficiency.

**Conclusions:**

The study showed that the G6PD deficient phenotype exists in Metehara even if the prevalence is not very high. G267 + 119C/T mutation is the predominant G6PD variant in this area. Therefore, malaria patient treatment using primaquine should be monitored closely for any adverse effects.

**Supplementary Information:**

The online version contains supplementary material available at 10.1186/s12936-022-04269-5.

## Background

Glucose-6-phosphate dehydrogenase (G6PD) is a house-keeping enzyme for all cells and particularly important for the integrity and functioning of red blood cells (RBCs). It catalyzes the production of nicotinamide adenine dinucleotide phosphate (NADPH) and provides the cell reduced form of glutathione, thus glutathione helps the erythrocytes to survive oxidative stress [1–4]. Glucose-6-phosphate dehydrogenase deficiency (G6PDd) is an X-linked genetic disorder caused by mutations in the *G6PD* gene on X-chromosome (Xq28) [2]. The X-linkage results in G6PDd of hemizygous males and homozygous females, while heterozygous females acquire two groups of RBCs with either normal or deficient G6PD activities [5, 6]. The mutations of *G6PD* gene results in protein variants with different levels of enzyme activity that are associated with a wide range of biochemical and clinical phenotypes. More than 200 mutations are identified and recognized as a cause of G6PDd, which affects over 400 million people worldwide [7–10]. According to World Health Organization (WHO) estimation, 7.5% of the world population are carriers of G6PDd and 2.9% are G6PD deficient. Although most variants have only slightly subnormal RBC survival, the Mediterranean variant renders the cells highly susceptible to oxidative stress [11].

Individuals with G6PDd could present a spectrum of disorders including atherosclerosis, cardiovascular disease [12], neonatal jaundice, acute massive haemolysis, renal failure, and chronic haemolytic anaemia induced by exposure to certain drugs, infections, fava beans, chemicals, and herbal medicines [10]. The 8-aminoquinolines, primaquine (PQ) and tafenoquine (TQ) have similar activities against the pre-erythrocyte stages of *Plasmodium* species in the liver [13, 14]. Both PQ and TQ metabolites can oxidize haemoglobin and generate excessive reactive oxygen species that can cause lethal acute haemolytic anaemia (AHA) in malaria patients with inherited G6PDd [15]. Patients having 30% or below of the normal G6PD activities are vulnerable to primaquine induced haemolysis when an increased dose (30 mg) of primaquine is used daily for shorter period [16]. The clinical dilemma associated with G6PDd and 8-aminoquinolines urges the health providers to either prescribe primaquine therapy with risk of acute haemolytic anaemia or withhold therapy with risk of advanced clinical symptoms and onward transmission initiated by *Plasmodium vivax* patients [17]. According to previous studies, the prevalence of G6PDd in Ethiopia nationally was 8.9% for G6PD A + [18] and 23.26% in Southern Ethiopia [19]. The most common G6PD mutations detected in Ethiopia were G6PDA + , G6PDA-, G267 + 119C/T and ChrX:154535443 [20].

To achieve the goal of malaria elimination, anti-malarial drugs that block transmission by killing gametocytes and reducing the liver stage hypnozoites of *P. vivax* are vital [16]. PQ is the only licensed drug that eradicate the gametocyte stages of all *Plasmodium* species and hypnozoites stages of *P. vivax* in infected hosts [20, 21]. A cohort study in Papua New Guinea indicates that 80% of *P. vivax* infections occurs by activating the incubation period of liver stage rather than new infections [22]. The WHO recommends mass screening of the population in regions where the prevalence of G6PDd is more than 3–5% before the administration of primaquine for elimination purpose [23, 24]. Before 1990, primaquine was used in Ethiopia for over a quarter of a century until it was removed from malaria treatment regimen, even though with no documented evidence of adverse effects. Currently, it has been re-incorporated into the treatment policy and targets the sexual and liver stages of *Plasmodium* species in malaria elimination strategy [21, 25].

Although the Metehara district is known to have an increased prevalence of malaria infection, there is limited information about the distribution of G6PDd. Therefore, this study aims to determine the prevalence of G6PDd and genotype of G6PD in the Metehara district of Ethiopia. Information of G6PDd in the population will scale-up the use of primaquine that can contribute to timely and successful elimination of malaria from the country.

## Methods

### Study design and sample collection

This study was conducted in Metehara Health Centre, Eastern Ethiopia. Metehara is an administrative town in Fentale Woreda, which is in the Great Rift Valley about 210 km east of the capital city, Addis Ababa (latitude and longitude coordinate: 8°540 N/39°550 E; average elevation is 947 m above sea level). Basaka lake and Awash river are major water bodies in the study area. Irrigation plantation activity that uses the nearby water sources for the industrial farming of sugar cane is suitable breeding site for *Anopheles* mosquitoes. The spread of malaria occurs all year round in this area, with the highest transmission season from September to November as well as from March to May [26]. The town has a population of 39,585 (19,397 male and 20,188 female) and there is one primary hospital and one government health centre (information from the Metehara town administration office, 2020). The 2019/2020 EFY report revealed that a total of 2,544 malaria cases diagnosed in Metehara town, of which *Plasmodium falciparum* and *P. vivax* accounted for 1809 (71%) and 735 (29%) of the total cases, respectively (information from the Metehara town administration health office). Individuals who visited the Metehara health centre with malaria clinical symptoms including fever, headache, fatigue, muscle and joint pain, chills, perspiration, and anorexia were recruited in a facility-based cross-sectional study from September 2020 to September 2021. Structured questionnaire was used to obtain socio-demographic and clinical information of all study participants.

A total of 498 study participants were selected using a quota sampling method during the study period. Individuals who came to the health facility for malaria diagnosis with at least two malaria symptoms were asked for consent/assent to participate in the study. Capillary and venous blood collection was performed by trained and experienced professionals [27, 28]. DBS samples were collected (Whatman, Maidstone, UK) and stored at − 20 °C freezer [21]. Malaria microscopy and malaria diagnosis using carestart™ malaria Pf/Pv (HRP2/pLDH) Ag combo RDT method were conducted at the study sites. All Giemsa stained slides were confirmed for its accuracy by a senior experienced microscopist.

### G6PD phenotype measurement and G6PD genotyping

Measurement of G6PD activity, Hb, G6PD/Hb ratio was done using careSTART™ G6PD biosensor (Access Bio, Seoul, Korea) following the manufacturer’s instruction for 498 clinical samples. The careSTART™ G6PD biosensor had variable performances: for samples with > 60% G6PD enzyme activity, sensitivity and specificity range from 53.7–100% and 64.9–98%; and for samples with < 30% G6PD enzyme activity, sensitivity and specificity range from 5.9–100% and 91.7–100%, respectively, against the standard quantitative G6PD enzyme activity test [29–33]. Briefly, for each sample, a G6PD test strip with two drops (20–30 μl) of finger-prick whole blood was inserted inside the biosensor at room temperature. The biosensor simultaneously indicates both the haemoglobin and G6PD readings within four minutes and was recorded automatically. A blank control was used to calibrate the G6PD biosensor to ensure the reading was zero before the next sample measurement. G6PD enzyme level was normalized by the concentration of haemoglobin (i.e. unit of G6PD enzyme per gram of haemoglobin, U/g Hb). The adjusted male median (AMM) G6PD activity, defined as the median G6PD activity of all male participants after excluding samples with less than 10% of the overall median activity, was calculated. For male study participants, G6PD deficient with < 30% of the AMM activity and G6PD normal with > 30% of the AMM activity. For female study participants, G6PD activity < 30%, 30–80%, and > 80% of the AMM activity are considered as G6PD deficient, intermediate, and normal, respectively [20, 34, 35]. Three PCR assays were conducted to determine the *G6PD* gene mutations of exon 4–11. G6PD genotyping was conducted following the protocol previously described [21] [20].

### Data analyses

All socio-demographic and clinical data was analysed using SPSS version-26. Mean, median, and standard deviation were computed for quantitative data. The relative contribution of independent variables for the outcome variables was assessed using logistic regression. A *p-value* of less than 0.05 was considered as significant association between G6PDd and each contributing factor (see Additional files 1, 2, 3, 4).

## Results

### Socio-demographic and clinical characteristics

Among the 498 study participants who have signs and symptoms of malaria 62% were male and 88.4% were ≥ 15 years (Table 1). The mean age of the study participants was 27.1 ± 12.8 years with a range of 4–75 years old. Among malaria suspected patients visiting Metehara Health Centre, 52% (259) were negative for all malaria species, 34.6% (172) were positive for *P. falciparum,* 9.2% (46) were positive for *P. vivax,* and 4.2% (21) were positive for both *P. falciparum* and *P. vivax* infections. More than half of the respondents had no history of previous malaria infection. Most of the study participants developed at least two clinical malaria symptoms, with headache and muscle/joint pain accounts 98.6% (491) and 90.4% (450), respectively (Table 1).

**Table 1:** Socio-demographic information and malaria symptoms of the study participants and its association with G6PD status, Metehara Health Center, Eastern Ethiopia, September 2020 to September 2021

Variables	G6PD status			Total N (%)
	Normal, n (%)	Intermediate, n (%)	Deficient, n (%)	
Sex				
Male	300 (97.1)	NA	9(2.9)	309(62.0)
Female	124 (65.6)	56 (29.6)	9 (4.8)	189 (38.0)
Age group (years)				
≤ 5	3 (100)	0	0	3 (0.6)
6–14	51 (92.7)	3 (5.5)	1 (1.8)	55 (11.0)
≥ 15	370 (84.1)	53 (12.0)	17 (3.9)	440 (88.4)
Residence				
Urban	282 (82.9)	46 (13.5)	12 (3.5)	340 (68.3)
Rural	142 (89.9)	10 (6.3)	6 (3.8)	158 (31.7)
History of malaria infection?				
No	261 (84.2)	43 (13.9)	6 (1.9)	310 (62.2)
Yes	163 (86.7)	13 (6.9)	12 (6.4)	188 (37.8)
Malaria status				
Negative	214 (82.6)	39 (15.1)	6 (2.3)	259 (52.0)
Positive	210 (87.9)	17 (7.1)	12 (5.0)	239 (48.0)
Headache				
No	5 (71.4)	1 (14.3)	1 (14.3)	7 (1.4)
Yes	419 (85.3)	55 (11.2)	17 (3.5)	491 (98.6)
Fatigue				
No	123 (87.2)	13 (9.2)	5 (3.5)	141 (28.3)
Yes	301 (84.3)	43 (12.0)	13 (3.6)	358 (71.7)
Muscle and joint pain				
No	40 (83.3)	6 (12.5)	2 (4.2)	48 (9.6)
Yes	384 (85.3)	50 (11.1)	16 (3.6)	450 (90.4)
Chills				
No	216 (85.4)	29 (11.5)	8 (3.2)	253 (50.8)
Yes	208 (84.9)	27 (11.0)	10 (4.1)	245 (49.2)
Perspiration				
No	224 (83.6)	36 (13.4)	8 (3.0)	268 (53.8)
Yes	200 (87.0)	20 (8.7)	10 (4.3)	230 (46.2)
Anorexia				
No	354 (85.7)	44 (10.7)	15 (3.6)	413 (82.9)
Yes	70 (82.4)	12 (14.1)	3 (3.5)	85 (17.1)

### Prevalence of G6PD deficiency and association with socio-demographic and clinical factors

**Fig. 1:**
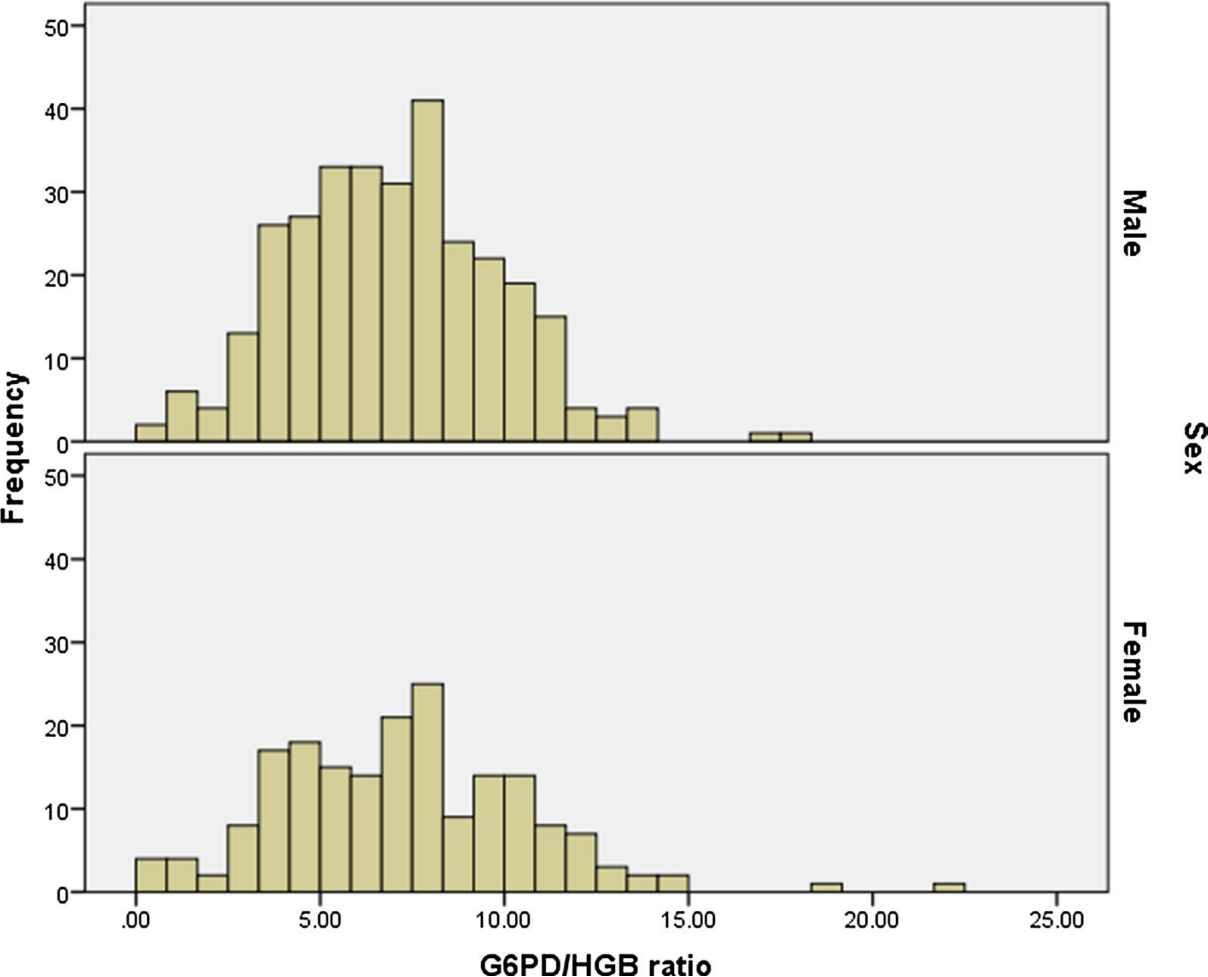
The G6PD enzyme activity distribution using careSTART™ G6PD biosensor assay among male and female study participants in Metehara Health Center, Ethiopia, 2021

**Fig. 2:**
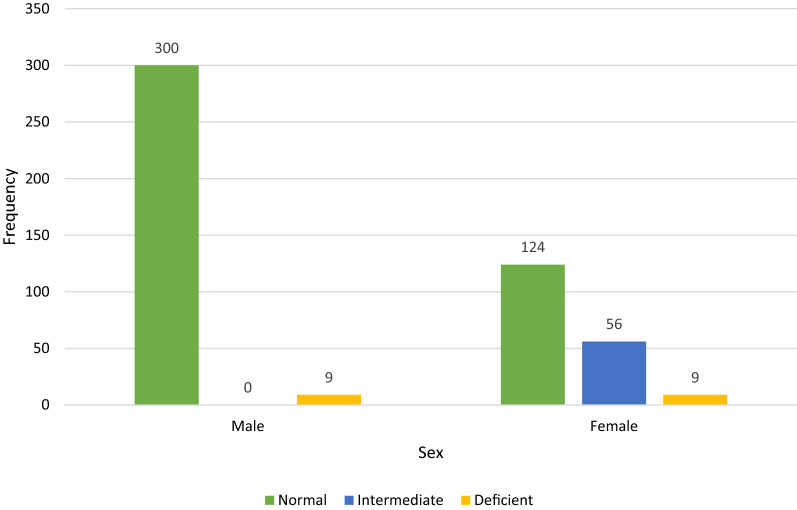
The proportion of patients with G6PD deficient (< 2.07 u/g Hb), intermediate (2.07–5.52 u/g Hb) and normal activities (> 5.52 u/g Hb) in Metehara Health Center, Eastern Ethiopia, from September 2020 to September 2021

Based on the adjusted male median (AMM) G6PD activity (6.9 U/g Hb), G6PD activities < 2.07 U/g Hb, 2.07–5.52 u/g Hb, and > 5.52 U/g Hb were indicated as G6PD deficient, intermediate, and normal, respectively. G6PD activities ranged from 0.2 to 17.9 U/g Hb in males and 0.2–22.3 U/g Hb in females (Fig. 1). The overall prevalence of G6PD deficiency was 3.6% (18/498), i.e., 2.9% (9/309) among males and 4.8% (9/189) among females. In addition, 66.7% (12/18) of G6PD deficient individuals were malaria positive. The females that showed intermediate (30–80% of AMM) G6PD activity were 11.2% (56/498) (Fig. 2).

Multivariate analysis result showed significant association of G6PD deficiency with sex, where females were three times more affected by G6PD deficiency than males (AOR = 3.0, 95% CI 1.1–8.6, *p-value* = 0.032). Similarly, Individuals with previous history of *Plasmodium* infection were more likely to be G6PD deficient than those who were not previously infected with malaria (AOR = 4.0, 95% CI 1.2–12.7, *p-value* = 0.02) (Table 2).

**Table 2:** Association between G6PD deficiency with different socio-demographic factors and clinical factors, Metehara Health Center, Eastern Ethiopia, September 2020 to September 2021

Characteristics	G6PDd status		COR (95% CI)	*p-value*	AOR (95% CI)	*p-value*
	Deficient	Normal				
Sex						
Male	9	300	1*		1*	
Female	9	180	1.7 (0.68–4.3)	0.29	3.0 (1.1–8.6)	0.032
Age group (years)						
≥ 15	16	423	1*		1*	
6–14	1	54	0.49 (0.06–3.8)	0.49	0.44(0.06–3.5)	0.44
≤ 5	1	3	8.8 (0.87–89.4)	0.07	35 (2.6–471)	0.007
Residence						
Urban	12	328	1*		1*	
Rural	6	152	1.08 (0.4–2.93)	0.88	0.9 (0.32–2.6)	0.88
History of malaria infection						
No	6	304	1*		1*	
Yes	12	176	3.5 (1.27–9.4)	0.015	4.0(1.2–12.7)	0.02
Malaria status						
Negative	6	253	1*		1*	
Positive	12	227	2.23 (0.82–6.0)	0.12	2.4 (0.73–7.7)	0.15

* Statistical reference

### G6PD mutations

A total of 17 DBS samples that were G6PD deficient (16) and intermediate (01) based on phenotypic measurement were sequenced for the *G6PD* gene exons 4–11. *G6PD* gene mutations were detected in 64.7% (11/17) of the samples. The G267 + 119C/T, GC was the most common mutation detected in the study population. From the 11 mutations, nine of them were single base substitution and the two [376, AT and ChrX: 154535443, GC] were polymorphic with G267 + 119C/T, GC mutation. Therefore, chX: 154535443 may have very limited impact on G6PD enzymatic activity, whereas the role of G267 + 119C/T requires further assessment. The predominant single mutation G267 + 119C/T was detected among 36.4% of male and 63.6% of female phenotypically G6PDd individuals. It was detected in 63.6% and 36.4% of malaria positive and malaria negative phenotypically G6PDd individuals, respectively (Table 3).

**Table 3:** Distribution of G6PD deficiency genetic variants with respect to sex, age group and malaria status of the study population, Metehara Health Center, Eastern Ethiopia, from September 2020 to September 2021

Characteristics	G6PD genotype					
	G267 + 119C/T		A376T		ChrX: 154535443	
	Wild type (%)	Mutant GC (%)	Wild type (%)	Mutant AT	Wild type (%)	Mutant GC (%)
Sex						
Male	1 (33.3)	4 (36.4)	4 (40)	0	5 (41.7)	0
Female	2 (66.7)	7 (63.6)	6 (60)	1	8 (58.3)	1 (100)
Malaria status						
Positive	1 (33.3)	7 (63.6)	7 (70)	0	8 (58.3)	0
Negative	2 (66.7)	4 (36.4)	3 (30)	1	5 (41.7)	1 (100)
Age group (years)						
≤ 5	0	1 (9)	1 (10)	0	1 (7.7)	0
5–14	0	1 (9)	1 (10)	0	1 (7.7)	0
≥ 15	3 (100)	9 (82)	8 (80)	1	11 (84.6)	1 (100)

## Discussion

In this study, phenotypic analysis of G6PD enzyme activity indicated that 3.6% (18/498) of the study participants had < 30% enzyme activity of the AMM, which is stated as G6PD deficiency; and 56 female patients had intermediate G6PD enzyme activity (30–80%) of AMM with heterozygous gene mutation. The G6PDd was slightly higher in females (4.8%) than males (2.8%). This result contradicts the assumption that females are anticipated to be less likely affected with G6PDd given the *G6PD* gene is located on the X-chromosomes. Males are hemizygous with only one copy of *G6PD* gene on X-chromosome, and thus can be either normal or G6PD deficient. By contrast, females have two copies of the *G6PD* gene on each X-chromosome, which can result in either homozygous normal, heterozygous intermediate, or homozygous deficient enzyme level [2]. Nguetse et al. [36] showed that 22% of males’ children are G6PDA- deficient while only 3% of females are G6PDA- deficient. The prevalence of G6PDd found in this study was slightly lower than a previous study conducted in seven sites of Ethiopia, which reported 5.4% (10/184) in males and 5.2% (7/136) in females [20] as G6PDd. Compared to other African countries such as Ghana, Gabon, and Kenya [36] with a G6PDd of 13% (36/278), this study revealed relatively lower G6PDd. The present finding is also lower than 7.3% (33/449) reported in Gambella, Southwest Ethiopia (8.6% in males and 6.3% in females) [37] and nation-wise (8.9% G6PDd) [18]. Nevertheless, the G6PDd in Metehara was higher than Jimma [38] and the community-based study in other parts of Ethiopia that showed relatively low levels of G6PD deficiency [21].

The X-linked nature of G6PD deficiency offers an advantage for female to be less likely affected by this enzyme disorder. The higher prevalence of G6PD deficiency observed among females compared to males in this study could be due to the less optimal performance of the G6PD biosensor, which merits further validation using spectrophotometric method. G6PD enzyme activity varies among nations, regions, and even local ethnic groups. The prevalence of G6PDd was slightly higher among Nuers (14.3%) and Anuak (12.0%) compared to the highlanders in Gambella region with no observed deficiency [37]. Malaria infection might impose selection for G6PD genetic mutation that results in higher prevalence of G6PDd in malaria endemic than non-endemic areas [21]. Metehara is one of the malaria endemic areas in Ethiopia with seasonal transmission where we observed higher G6PDd prevalence than previous community-based studies in different settings but lower than similar studies in stable endemic areas like Gambella [37].

Although G6PD deficiency was postulated to have a protective effect against malaria infection, G6PD deficient individuals can still be infected with *Plasmodium* species [39, 40]. In this study, 66.7% (12/18) of G6PD deficient individuals were malaria positive. Similar study in Upper Myanmar showed that among 50 malaria patients with G6PD variants, 25 (50.0%), 17 (34.0%), and 8 (16.0%) were infected with *P. falciparum, P. vivax*, and co-infections with both *P. falciparum* and *P. vivax*, respectively [41]. G6PDd was significantly higher in the malaria positive patients than malaria negative patients [37]. Malaria infections showed marginal but not statistically significant association with G6PDd, while sex and history of malaria infection were significantly associated with G6PDd. The prevalence of G6PDd among females was three times higher than in males and was four times higher in previously malaria infected patients than those without previous infection. While the prevalence of G6PDd was higher in males than females in Gambella Hospital, Ethiopia [37], no significant association of G6PDd with sex and malaria infection was found broadly across Ethiopia [20]. This variation in associations of independent factors with the prevalence of G6PDd might be due to medical status of the study participants. It is important to note that this study included only participants with malaria sign and symptoms. Variations in host immune responses and other medical conditions among the study patients may influence the prevalence of G6PD deficiency.

Based on molecular sequencing, mutations were detected in G267 + 119C/T, A376T and ChrX: 154535443 with G267 + 119C/T being most prevalent. Although previous studies in Ethiopia reported the presence of A376G, G202A, C563T, G1116A and 485 + 37, these mutations were not identified in this study. Contrary to this study, 13% of the study participants showed G6PDA- (G202A) genotype in Brazzaville, Republic of Congo [11], 12.5% (39/311) depicted G6PDA + (A376G) in Eritrea [42]. Several studies in different localities of Ethiopia showed that G6PDA + (A376G) was the only mutation observed in 8.9% of the studied population [18], and 23.26% (20/86) G6PDA + mutations were detected in Southwestern Ethiopia [19]. The G202A mutation was also detected in 3.5% of the individuals [21] and in another study, G6PDA + mutation was detected in 6.1% (21/344) of individuals, G267 + 119C/T and G1116A mutations in 1.2% (4) and 1.2% (4) individuals, respectively [20]. No mutation was detected in Shewa Robit [20]. In the Oromia region, out of the 34 low enzyme activity samples, only one G6PDA + and one G445A mutations were identified [38].

Previous studies found only few variants such as G6PDA + in different parts of Ethiopia [18, 20], G267 + 119C/T and G1116A in the southern parts of the county [19, 20], and one mutation at position 445GA in Jimma [38]. This study identified a new genotype, A376T mutation, in addition to those others previously documented. The A376T mutation represents the substitution of adenine by thyamine, resulting in amino acid replacement of 126 Asn with Tyr. While this mutation is new to Ethiopia, it was previously found in Mexico [43]. There were six samples with no detected mutations despite showing low enzyme activities. In another study, one sample with low G6PD enzyme activity had no G6PD mutation [20]. It is possible that the mutations observed in A376G, G267 + 119C/T, and G1116A were not associated with low G6PD activity [20]. The occurrence of new genetic variants in such a small-scale study implies the need of a large scale G6PDd epidemiological survey to characterize the full array of *G6PD* genetic variants across the country as well as the functional significance of the variants. The low performance of the careSTART™ G6PD biosensor may also explain samples of low enzyme activity but with no observed gene mutations. Future analysis of G6PD enzyme activity by the careSTART™ G6PD biosensor would require validation using spectrophotometric method. Molecular sequencing of the G6PD enzyme would need to be done for all phenotypically G6PD normal and intermediate samples to investigate thoroughly the phenotype and genotype association. The lack of association of G6PD enzyme activities with respective genotypes advocates the need for further verification with genome-wide association testing and large sample size.

## Conclusion

The prevalence of G6PD deficiency is low in the Metehara district based on phenotypic measurement. Such prevalence was significantly associated with sex and previous malaria infection history. G267 + 119C/T was the predominant genetic variant detected among the study participants. Thus, it is recommended to treat malaria patients with primaquine under close supervision and cautious follow-up for any haemolytic complications in the Metehara district. The presence of the new mutation in Ethiopia offer insights into the presence of various *G6PD* genetic variants in the country. Future study should aim for national-wide epidemiologic study of G6PD deficiency in Ethiopia.

## Supplementary Information

**Additional file 1: **Information sheet.

**Additional file 2: **Consent and assent.

**Additional file 3: **Questionnaire.

**Additional file 4: **Laboratory procedures.

## Data Availability

The data produced in the study is included in the main manuscript and the rest are available upon reasonable request from the corresponding author.

## Abbreviations

G6PD: Glucose-6-phosphate dehydrogenase
G6PDd: Glucose-6-phosphate dehydrogenase deficiency
NADPH: Nicotinamide adenine dinucleotide phosphate
RBCs: Red blood cells
PQ: Primaquine
TQ: Tafenoquine
AMM: Adjusted male median
AMM: Adjusted male median, the median of all G6PD enzyme activity of male study participants by excluding values < 10% of the median of the whole participants
Deficient: The G6PD enzyme activity of both male and female study participants which is less than 30% of AMM
Intermediate: The G6PD enzyme activity of female study participants which are between 30 and 80% of the AMM
Non-deficient: The G6PD enzyme activity which are greater than 30% of AMM for male study participants and greater than 80% of AMM for female study participants
History of malaria infection: A person who had ever been infected with malaria in the previous time of his life

## References

[1] Nkhoma ET, Poole C, Vannappagari V, Hall SA, Beutler E (2009). The global prevalence of glucose-6-phosphate dehydrogenase deficiency: a systematic review and meta-analysis. Blood Cells Mol Dis.

[2] Cappellini MD, Fiorelli G (2008). Glucose-6-phosphate dehydrogenase deficiency. Lancet.

[3] Bharti RS, Vashisht K, Ahmed N, Nayak A, Pande V, Mishra N (2020). First report of glucose-6-phosphate dehydrogenase (G6PD) variants (Mahidol and Acores) from malaria-endemic regions of northeast India and their functional evaluations in silico. Acta Trop.

[4] Peters AL, van Noorden CJ (2017). Single cell cytochemistry illustrated by the demonstration of glucose-6-phosphate dehydrogenase deficiency in erythrocytes. Methods Mol Biol.

[5] Valencia SH, Ocampo ID, Arce-Plata MI, Recht J, Arévalo-Herrera M (2016). Glucose-6-phosphate dehydrogenase deficiency prevalence and genetic variants in malaria endemic areas of Colombia. Malar J.

[6] Manganelli G, Masullo U, Passarelli S, Filosa S (2013). Glucose-6-phosphate dehydrogenase deficiency: disadvantages and possible benefits. Cardiovasc Hematol Disord Drug Targets.

[7] Li Q, Yang F, Liu R, Luo L, Yang Y, Zhang L (2015). Prevalence and molecular characterization of glucose-6-phosphate dehydrogenase deficiency at the China-Myanmar border. PLoS ONE.

[8] Ouattara AK, Yameogo P, Diarra B, Obiri-Yeboah D, Yonli A, Compaore TR (2016). Molecular heterogeneity of glucose-6-phosphate dehydrogenase deficiency in Burkina Faso: G-6-PD betica selma and santamaria in people with symptomatic malaria in Ouagadougou. Mediterr J Hematol Infect Dis.

[9] Francis RO, Jhang JS, Pham HP, Hod EA, Zimring JC, Spitalnik SL (2013). Glucose-6-phosphate dehydrogenase deficiency in transfusion medicine: the unknown risks. Vox Sang.

[10] Gunawardena S, Kapilananda G, Samarakoon D, Maddevithana S, Wijesundera S, Goonaratne LV (2017). Prevalence of G6PD deficiency in selected populations from two previously high malaria endemic areas of Sri Lanka. PLoS ONE.

[11] Gueye NSG, Peko SM, Nderu D, Koukouikila-Koussounda F, Vouvoungui C, Kobawila SC (2019). An update on glucose-6-phosphate dehydrogenase deficiency in children from Brazzaville, Republic of Congo. Malar J.

[12] Thomas JE, Kang S, Wyatt CJ, Kim FS, Mangelsdorff AD, Weigel FK (2018). Glucose-6-Phosphate dehydrogenase deficiency is associated with cardiovascular disease in US Military centers. Tex Heart Inst J.

[13] Ahmad SS, Rahi M, Sharma A (2021). Relapses of *Plasmodium vivax* malaria threaten disease elimination: time to deploy tafenoquine in India?. BMJ Glob Health.

[14] Cui Y, Zhang L, Xia Z, Zhou H, Huang F (2021). Epidemiological characterization of imported recurrent *Plasmodium vivax* and *Plasmodium ovale* in China, 2013–2020. Infect Dis Poverty.

[15] Baird JK, Surjadjaja C (2011). Consideration of ethics in primaquine therapy against malaria transmission. Trends Parasitol.

[16] Ghimire P, Singh N, Ortega L, Rijal KR, Adhikari B, Thakur GD (2017). Glucose-6-phosphate dehydrogenase deficiency in people living in malaria endemic districts of Nepal. Malar J.

[17] Baird JK (2021). Basic research of *Plasmodium vivax* biology: enabling its management as a clinical and public health problem. Front Cell Infect Microbiol.

[18] Assefa A, Ali A, Deressa W, Tsegaye W, Abebe G, Sime H (2018). Glucose-6-phosphate dehydrogenase (G6PD) deficiency in Ethiopia: absence of common African and Mediterranean allelic variants in a nationwide study. Malar J.

[19] Carter TE, Mekonnen SK, Lopez K, Bonnell V, Damodaran L, Aseffa A (2018). Glucose-6-phosphate dehydrogenase deficiency genetic variants in malaria patients in Southwestern Ethiopia. Am J Trop Med Hyg.

[20] Lo E, Zhong D, Raya B, Pestana K, Koepfli C, Lee M-C (2019). Prevalence and distribution of G6PD deficiency: implication for the use of primaquine in malaria treatment in Ethiopia. Malar J.

[21] Shitaye G, Gadisa E, Grignard L, Shumie G, Chali W, Menberu T (2018). Low and heterogeneous prevalence of glucose-6-phosphate dehydrogenase deficiency in different settings in Ethiopia using phenotyping and genotyping approaches. Malar J.

[22] Robinson LJ, Wampfler R, Betuela I, Karl S, White MT, Li Wai Suen CS (2015). Strategies for understanding and reducing the *Plasmodium vivax* and *Plasmodium ovale* hypnozoite reservoir in Papua New Guinean children: a randomised placebo-controlled trial and mathematical model. PLoS Med..

[23] Thielemans L, Gornsawun G, Hanboonkunupakarn B, Paw MK, Porn P, Moo PK (2018). Diagnostic performances of the fluorescent spot test for G6PD deficiency in newborns along the Thailand-Myanmar border: a cohort study. Wellcome Open Res.

[24] Working Group (1989). Glucose-6-phosphate dehydrogenase deficiency. Bull World Health Organ.

[25] WHO (2017). A framework for malaria elimination.

[26] Nega D, Assefa A, Mohamed H, Solomon H, Woyessa A, Assefa Y (2016). Therapeutic efficacy of artemether-lumefantrine (Coartem®) in treating uncomplicated *P.*
*falciparum* malaria in Metehara, Eastern Ethiopia: regulatory clinical study. PLoS ONE.

[27] WHO (2010). Guidelines on drawing blood: best practices in phlebotomy.

[28] WHO (2014). Point-of-care G6PD testing to support safe use of primaquine for the treatment of vivax malaria. WHO evidence review group meeting report.

[29] Pengboon P, Thamwarokun A, Changsri K, Kaset C, Chomean S (2019). Evaluation of quantitative biosensor for glucose-6-phosphate dehydrogenase activity detection. PLoS ONE.

[30] Djigo OKM, Khalef YO, Salem MSOA, Gomez N, Basco L, Briolant S (2021). Assessment of CareStart G6PD rapid diagnostic test and CareStart G6PD biosensor in Mauritania. Infect Dis Poverty.

[31] Ley B, Alam MS, O’Donnell JJ, Hossain MS, Kibria MG, Jahan N (2017). A comparison of three quantitative methods to estimate G6PD activity in the Chittagong Hill Tracts, Bangladesh. PLoS ONE.

[32] Alam MS, Kibria MG, Jahan N, Thriemer K, Hossain MS, Douglas NM (2018). Field evaluation of quantitative point of care diagnostics to measure glucose-6-phosphate dehydrogenase activity. PLoS ONE.

[33] Weppelmann TA, von Fricken ME, Wilfong TD, Aguenza E, Philippe TT, Okech BA (2017). Field trial of the CareStart biosensor analyzer for the determination of glucose-6-phosphate dehydrogenase activity in Haiti. Am J Trop Med Hyg.

[34] Ley B, Bancone G, Von Seidlein L, Thriemer K, Richards JS, Domingo GJ (2017). Methods for the field evaluation of quantitative G6PD diagnostics: a review. Malar J.

[35] Zobrist S, Brito M, Garbin E, Monteiro WM, Clementino Freitas S, Macedo M (2021). Evaluation of a point-of-care diagnostic to identify glucose-6-phosphate dehydrogenase deficiency in Brazil. PLoS Negl Trop Dis.

[36] Nguetse CN, Meyer CG, Adegnika AA, Agbenyega T, Ogutu BR, Kremsner PG (2016). Glucose-6-phosphate dehydrogenase deficiency and reduced haemoglobin levels in African children with severe malaria. Malar J.

[37] Tsegaye A, Golassa L, Mamo H, Erko B (2014). Glucose-6-phosphate dehydrogenase deficiency among malaria suspects attending Gambella hospital, southwest Ethiopia. Malar J.

[38] Kießling N, Brintrup J, Zeynudin A, Abduselam N, Götz S, Mack M (2018). Glucose-6-phosphate dehydrogenase activity measured by spectrophotometry and associated genetic variants from the Oromiya zone, Ethiopia. Malar J.

[39] Amoah LE, Asare KK, Dickson D, Abankua J, Busayo A, Bredu D (2021). Genotypic glucose-6-phosphate dehydrogenase (G6PD) deficiency protects against *Plasmodium falciparum* infection in individuals living in Ghana. PLoS ONE.

[40] Galatas B, Mabote L, Simone W, Matambisso G, Nhamussua L, Del Mar M-P (2017). Heterogeneity of G6PD deficiency prevalence in Mozambique: a school-based cross-sectional survey in three different regions. Malar J.

[41] Lee J, Im Kim T, Kang J-M, Jun H, Le HG, Thai TL (2018). Prevalence of glucose-6-phosphate dehydrogenase (G6PD) deficiency among malaria patients in Upper Myanmar. BMC Infect Dis.

[42] Tseghereda YG, Nganga JK, Kimang’a AN, Mehari TH, Weldemichael YG (2018). Glucose-6-phosphate dehydrogenase deficiency allelic variants and their prevalence in malaria patients in Eritrea. Pan Afr Med J.

[43] Gómez-Manzo S, Marcial-Quino J, Vanoye-Carlo A, Serrano-Posada H, Ortega-Cuellar D, González-Valdez A (2016). Glucose-6-phosphate dehydrogenase: update and analysis of new mutations around the world. Int J Mol Sci.

