# Supplementary material for: Prevalence of G6PD deficiency and distribution of its genetic variants among malaria-suspected patients visiting Metehara health centre, Eastern Ethiopia
Source: Malar J. 2022 Sep 8;21:260. doi: 10.1186/s12936-022-04269-5 (PMC9461287; doi:10.1186/s12936-022-04269-5)
Supplement: Supplementary file 1 — Additional file 1: Information sheet. [file 12936_2022_4269_MOESM1_ESM.docx]

## Information sheet (English version)

Addis Ababa University Postgraduate schedule

**PI**: Tassew Tefera

**Name of organization**: Addis Ababa University College of Health Science, School of Medicine, Department of Microbiology, Immunology and Parasitology information sheet.

**Title**: Prevalence of Glucose-6-phosphate dehydrogenase deficiency in Metehara District (Eastern Ethiopia) among attendants of selected health facilities suspected to have plasmodium infection.

**Aim**: To determine the magnitude of Glucose-6-phosphate dehydrogenase deficiency among attendants of selected health facilities in Metehara district, Ethiopia

**Duration**: For the purpose of G6PD rapid testing and blood sample collection you will spend only 10 to 20 minutes. The questionnaire will be filled and the consent form is signed.

**Procedure to be followed**: For this study to be successful we need your participation. If you are voluntary to participate, you are expected to understand and sign the informed consent. Socio demographic information is important and will be taken for the study. Venous blood and capillary blood sample will be collected and laboratory investigation will be done at onsite during specimen collection and EPHI after collection.

**Risk**: There is only minimal risk associated with sample collection and your time.

**Expected benefits**: As a participant of the study you are expected to give capillary and venous blood sampleand we will measurethe quantity of G6PD, Hemoglobin and G6PD/Hgb ratio using careSTART POCT S1 and Spectrophotometer methods. The genetic variants of G6PD deficient gene mutation will be performed by molecular method. The result will be discussed with the responsible physician but your personal information will not be disclosed to anyone. Only identification code will be used.

**Confidentiality**: All information that you give and the results from your specimen will be used for this study only. Limited number of professionals will have access to the information. All the information will be encoded in a computer and password protected.

**Right**: Refusal to participate in the study involves no penalty or loss of benefit to which you are otherwise entitled and participation is voluntary. You have the right to withhold information, decline to cooperate in the study and refuse provision of specimen.

**Approval**: This research project has got ethical clearance from the department research and ethics review committee (DRERC) of Addis Ababa University College of Health Science, School of Medicine, Department of Microbiology, Immunology and Parasitology and institutional review board of EPHI.

**Whom to contact**: If you have any question about this study you can communicate through the following address.

- Addis Ababa University College of Health Science School of Medicine, Department of Microbiology, Immunology and Parasitology
- Tel. --------------------- Fax.------------------- Email----------------------
- Principal Investigator : Tassew Tefera Phone : 0922406465

E-mail : [tassewtefera@gmail.com](mailto:tassewtefera@gmail.com)

## Information sheet (Amharic version)

**አዲስ አበባ ዩኒቨርሲቲ የድህረ-ምረቃ ፕሮግራም**

**ጥናቱን የሚሰራዉ ሰዉ ስም፡** ጣሰዉ ተፈራ

**ጥናቱን የሚያሰራዉ ተቁዋም፡** አዲስ አበባ ዩኒቨርሲቲ የጤና ሳይንስ ኮሌጅ የህክምና ሳይንስ ት/ቤት የማይክሮባዮሎጂ፤ ኢሚዩኖሎጂ እና ፓራሳይቶሎጂ ትምህርት ክፍል

**የጥናቱ ርዕስ፡** የ”Glucose-6-phosphate dehydrogenase” እጥረት ስርጭት በመተሃራ ጤና ጣቢያ በሚል ርዕስ ለሚደረገዉ ጥናት የተዘጋጀ መረጃ በአዲስ አበባ ዩኒቨርሲቲ በ”ፓራሳይቶሎጂ” የማስተርስ ዲግሪ ተማሪ የመመረቂያ ጥናት ላይ እንዲሳተፉ ተጋብዘዋል፡፡ እባክዎ በዚህ ጥናት ለመሳተፍ ከመስማማትዎ በፊት ከዚህ ቀጥሎ የሚገኘዉን ምንባብ በጥሞና ያንብቡና ግልፅ ያልሆነዉን ይጠይቁ፡፡

**የጥናቱ ዓላማ፡** የ”Glucose-6-phosphate dehydrogenase” እጥረት የስርጭት መጠንን ማጥናት

**እዚህ የሚቆዩበት ጊዜ፡** የተሰጠዎትን መጠይቅ ከሞሉና ከፈረሙ በኋላ ለጥናቱ የሚያስፈልገዉን የደም ናሙና በመስጠት ሂደቱን ይጨርሳሉ፡፡ ለዚህም ተግባር ከ 10 እስከ 20 ደቂቃ ብቻ ያጠፋሉ፡፡

**በዚህ ጥናት ሲሳተፉ የሚፈፅሟቸዉ ተግባራት፡** ለዚህ ጥናት መሳካትና ዉጤታማነት የእርስዎ አስተዋፅዖ በጣም ከፍተኛ ሲሆን ለመሳተፍ ፈቃደኛ በመሆንዎ እያመሰገንን፣ በጥናቱ ከመሳተፍዎ በፊት የፈቃደኝነት ዉሉን በደንብ አንብበዉ በመረዳት መፈረም አለብዎት፡፡ ዉሉን ከፈረሙ በኋላ በቃለ መጠይቁ ላይ የቀረበዉን ርስዎን የሚመለከት አጠቃላይ መረጃ በጥንቃቄ ከሞሉ በኋላ በሰለጠኑ ባለሙያዎች ከጣትዎ ላይ ሁለት ጠብታ ደም ተወስዶ የ G6PD እና Hemoglobin መጠን ይለካልዎታል፤ ለሞለኪዩላር ምርመራ DBS ናሙና ይሰበሰባል፡፡ በተጨማሪ 4 ሚሜ ደም ከክንድዎ ላይ በመርፌ ተቀድቶ ወደ ኢትዮጵያ ህብረተሰብ ጤና ኢንስትቲዩት ላቦራቶሪ ይወሰዳል፡፡

**በዚህ ጥናት መሳተፍ የሚያስከትለዉ ጉዳት፡** በጥናቱ መሳተፍ ናሙና ለመስጠት ከሚየጠፉት ጊዜ እና የደም ናሙና ሲወሰድ ከሚሰማዎት ቀላል ህመም ዉጪ ምንም ዓይነት ጉዳት አያስከትልም፡፡

**በዚህ ጥናት መሳተፍ የሚያስገኛቸዉ ጥቅሞች፡** የጥናቱ ተሳታፊ በመሆንዎ የሚያመጡት የደም ናሙና ጥራቱን በጠበቀ የአሰራር ሂደትና ልምድ ባላቸዉ ባለሙያዎች ሦስት ዓይነት የተሻሻሉ የላቦራቶሪ የምርመራ ዘዴዎችን በመጠቀም የG6PD and Hemoglobin ምርመራ ይሰራልዎታል፡፡

**የመረጃ ሚስጥራዊነት:** እርስዎ የሚሰጡት መረጃና ከደም ናሙናዉ የሚገኘዉ ዉጤት ለዚህ ጥናት ዓላማ ብቻ ይዉላል፡፡ የምርመራ ዉጤትዎንም ለሚመለከተዉ ሃኪምዎ ብቻ በማሳዎቅ አስፈላጊዉን ህክምና እንዲያገኙ እናደርጋለን፡፡ በስም ምትክ የሚስጥር ቁጥር ስለምንጠቀም ማንኛዉንም ዓይነት መረጃዎን ከሚመለከተዉ አካል ዉጪ ያለርስዎ ፈቃድ አናሳዉቅም፡፡

**የጥናቱ ተሳታፊ መብት**፡ በዚህ ጥናት መሳተፍ የሚቻለዉ በራስ ተነሳሽነትና በሙሉ ፈቃደኝነት በመሆኑ፤ በማንኛዉም ጊዜና ሁኔታ መሳተፍ አልፈልግም ብሎ መተዉ ይቻላል፡፡ ጥናቱን አልሳተፍም ብሎ በመተዉ ምክንያት የሚደርስ ምንም ዓይነት ቅጣት፣ ኪሳራ እና ያልተገባ ዉንጀላ ወይም ነቀፌታ የለም፡፡ ፈቃደኛ ካልሆኑ መረጃዎትን የመደበቅ ወይም ያለመናገር፣ በጥናቱ ያለመሳተፍ አናም ለጥናቱ የሚያስፈልገዉን የደም ናሙና ያለመስጠት ሙሉ መብት አለዎት፡፡

**ስለ ጥናቱ ማረጋገጫ፡** ይህ ጥናት ከአዲስ አበባ ዩኒቨርሲቲ የጤና ሳይንስ ኮሌጅ ህክምና ሳይንስ ት/ቤት የማይክሮባዮሎጂ፤ ኢሚዩኖሎጂ እና ፓራሳይቶሎጂ ትምህርት ክፍል የምርምርና ህጋዊነት ኮሚቴ እዉቅናና ፈቃድ አግኝቷል፡፡ እንዲሁም ጥናቱ በሚሰራበት በመተሃራ ከተማ ጤና ተቐማት የበላይ ሀላፊዎችን በማስፈቀድ የሚሰራ ጥናት ነዉ፡፡

**ጥያቄ ቢኖረኝ/ችግር ቢያጋጥመኝ ምን ማድረግ እችላለሁ**፡- ጥናቱን የተመለከተ ማንኛዉም ዓይነት ጥያቄ ካለዎት በሚከተሉት አድራሻዎች በመጠቀም መጠየቅ ይችላሉ፤

አዲስ አበባ ዩኒቨርሲቲ ጤና ሳይንስ ኮሌጅ የህክምና ሳይንስ ት/ቤት የማይክሮባዮሎጂ፤ ኢሚዩኖሎጂ እና ፓራሳይቶሎጂ ትምህርት ክፍል

ስልክቁጥር፡ ----------------------- ፋክስ፡ ----------------------------- ኢ-ሜይል፡ ------------------------

ጥናቱን የሚሰራዉ፡ ጣሰዉ ተፈራ

ስልክ ቁጥር፡ 0922406465 ኢ-ሜይል፡[tassewtefera@gmail.com](mailto:tassewtefera@gmail.com)
