# Supplementary material for: Prevalence of G6PD deficiency and distribution of its genetic variants among malaria-suspected patients visiting Metehara health centre, Eastern Ethiopia
Source: Malar J. 2022 Sep 8;21:260. doi: 10.1186/s12936-022-04269-5 (PMC9461287; doi:10.1186/s12936-022-04269-5)
Supplement: Supplementary file 2 — Additional file 2: Consent and assent. [file 12936_2022_4269_MOESM2_ESM.docx]

**Consent form for the study participants (English Version)**

Code No. ----------------------

Name of the participant ------------------------

I have been informed about the study which is aimed in determining the magnitude of Glucose-6-phosphate dehydrogenase deficiency among attendants of selected health facilities in Metehara, Ethiopia. For this study blood sample is required. The aim and possible risk of the study were explained to me well. I have also informed that all the information contained in questionnaire is to be kept confidential. Moreover, I have been informed the rights to withdraw from study. It is therefore with full understanding I gave the informed consent voluntarily to the researcher to use my information and specimen for this study.

Participant’s signature/Finger print ---------------------------

Name of data collector ------------------------------------ Sign --------------------- Date ---------------

Please contact us for any question or problems you may encounter during the study

Principal investigator: Tassew Tefera

Phone- 0922406465

E-mail:[tassewtefera@gmail.com](mailto:tassewtefera@gmail.com)

**Assent form for the study participants (English Version)**

Code No. ----------------------

Name of study participant ---------------------------

Name of the participant’s family or Guardian ------------------------

I have been informed about the study which is aimed in determining the magnitude of Glucose-6-phosphate dehydrogenase deficiency among attendants of selected health facilities in Metehara district, Ethiopia. For this study blood sample is required. The aim and possible risk of the study were explained to me well. I have also informed that all the information contained in questionnaire is to be kept confidential. Moreover, I have been informed the rights to withdraw from study. The study participant mentioned above who is not able to give the consent himself because he/she is younger than 18 years not allowed deciding on him/her. It is therefore with full understanding; by taking a full responsibility I gave my assent voluntarily to the researcher to use his/her information and specimen for this study.

Participant’s signature/Finger print ---------------------------

Name of data collector ------------------------------------ Sign --------------------- Date ---------------

Please contact us for any question or problems you may encounter during the study

Principal investigator: Tassew Tefera

Phone- 0922406465

E-mail:[tassewtefera@gmail.com](mailto:tassewtefera@gmail.com)

## Consent form for the study participants (Amharic Version)

የጥናቱ ተሳታፊዎች የስምምነት ቅጽ

የሚስጥር ቁጥር ----------------------

የተሳታፊዉ ስም ------------------------

እኔ ከዚህ በላይ ስሜ የተጠቀሰዉ የጥናቱ ተሳታፊ “Glucose-6-phosphate dehydrogenase እጥረት ስርጭት በመተሃራ ጤና ጣቢያ” በሚል ርዕስ ስለሚሰራዉ የምርምር ስራ አስፈላጊዉ መረጃ ሁሉ ተነግሮኛል፡፡ ለዚህ ጥናት የደም ናሙና መስጠት እንዳለብኝ፣ የምርምሩ ዓላማ ምን እንደሆነ እና በጥናቱ ምክንያት ሊከሰቱ የሚችሉ ጉዳቶች በዝርዝር ማብራሪያ ተሰጥቶኝ ተረድቻለሁ፡፡ በተጨማሪም ማንኛዉም ዓይነት ለዚህ ጥናት የሰጠሁት መረጃዬ በሚስጥር ተጠብቆ እንደሚያዝልኝ እና በፈለግሁት ግዜና ሁኔታ በጥናቱ መሳተፍ ካልፈለግሁ ማቋረጥ መብቴ እንደሆነ በግልፅ ተነግሮኛል፡፡ ስለዚህ የተሰጠኝን መረጃ መሰረት በማድረግ እና ዓላማዉንም በመረዳት በፈቃዴ የምሰጠዉን እኔን የሚመለከት መረጃ እና የደም ናሙና ለምርምር አገልግሎቱ እንዲያዉሉት ተስማምቼ መፍቀዴን በፊርማዬ አረጋግጠለሁ፡፡

የተሳታፊዉ ፊርማ ---------------------------

የመረጃ ሰብሳቢዉ ስም --------------------------- ፊርማ --------------------- ቀን -------------------

ማንኛዉም ችግር ካጋጠመዎት በሚቀጥለዉ አድራሻዎች ያሳዉቁን

የጥናቱ ባለሙያ፡ ጣሰዉ ተፈራ

ስልክ ቁጥር፡ 0922406465

ኢ-ሜይል፡ [tassewtefera@gmail.com](mailto:tassewtefera@gmail.com)

## Assent form for the study participants (Amharic Version)

የጥናቱ ተሳታፊ ለሆኑ ህፃናት ወላጅ/አሳዳጊ የስምምነት ቅጽ

የሚስጥር ቁጥር ----------------------

የተሳታፊዉ ስም ------------------------

የተሳታፊዉ ወላጅ/አሳዳጊ ስም --------------------------

እኔ ከዚህ በላይ ስሜ የተጠቀሰዉ የጥናቱ ተሳታፊ ህፃን ወላጅ/አሳዳጊ ቤተሰብ “Glucose-6-phosphate dehydrogenase እጥረት ስርጭት በመተሃራ ጤና ጣቢያ” በሚል ርዕስ ስለሚሰራዉ የምርምር ስራ አስፈላጊዉ መረጃ ሁሉ ተነግሮኛል፡፡ ለዚህ ጥናት የደም ናሙና መስጠት እንዳለብኝ፣ የምርምሩ ዓላማ ምን እንደሆነ እና በጥናቱ ምክንያት ሊከሰቱ የሚችሉ ጉዳቶች በዝርዝር ማብራሪያ ተሰጥቶኝ ተረድቻለሁ፡፡ በተጨማሪም ማንኛዉም ዓይነት ለዚህ ጥናት የሰጠሁት መረጃዬ በሚስጥር ተጠብቆ እንደሚያዝልኝ እና በፈለግሁት ግዜና ሁኔታ በጥናቱ መሳተፍ ካልፈለግሁ ማቋረጥ መብቴ እንደሆነ በግልፅ ተነግሮኛል፡፡ ከላይ በስም የተጠቀሰዉ የጥናቱ ተሳታፊ ከ18 ዓመት እድሜ በታች በመሆኑና በራሱ ፈቃድ ለመሳተፍ ስምምነት መፈራረም ስለማይችል እኔ የእርሱ ወላጅ/አሳዳጊ ቤተሰብ በመሆኔ የተሰጠኝን መረጃ መሰረት በማድረግ እና ዓላማዉንም በመረዳት በፈቃዴ የምሰጠዉን ልጄን የሚመለከት መረጃ እና የደም ናሙና ለምርምር አገልግሎቱ እነዲያዉሉት ተስማምቼአለሁ፡፡

የተሳታፊዉ ፊርማ ------------------------------

የመረጃ ሰብሳቢዉ ስም --------------------------- ፊርማ --------------------- ቀን -------------------

ማንኛዉም ችግር ካጋጠመዎት በሚቀጥለዉ አድራሻዎች ያሳዉቁን

የጥናቱ ባለሙያ፡ ጣሰዉተፈራ ስልክ ቁጥር፡ 0922406465

ኢ-ሜይል፡ [tassewtefera@gmail.com](mailto:tassewtefera@gmail.com)
