# Supplementary material for: Prevalence of G6PD deficiency and distribution of its genetic variants among malaria-suspected patients visiting Metehara health centre, Eastern Ethiopia
Source: Malar J. 2022 Sep 8;21:260. doi: 10.1186/s12936-022-04269-5 (PMC9461287; doi:10.1186/s12936-022-04269-5)
Supplement: Supplementary file 3 — Additional file 3: Questionnaire. [file 12936_2022_4269_MOESM3_ESM.docx]

## Questionnaire (English version)

This questionnaire is prepared by Addis Ababa University, College of health science, Department of Medical Microbiology, Immunology and Parasitology graduate program student.

I thanks gratefully for your agreement to participate in this study. Now I am going to ask you interview questions and the interview is about general socio-demographic characteristics and clinical data. All of the answers you provide in this study will be kept confidential. The information you give me is very essential for this study. Therefore, I politely ask you to give me the right response.

Part I. Socio-demographic and Clinical information

1. Code no. --------------- Age…………… Sex: Male Female
2. Address: Urban Rural
3. Hemoglobin ---------------, G6PD---------------------, G6PD/Hgb ratio------------------
4. Have you ever been infected with malaria?
5. Yes B. No
6. If yes for quest. 4, did you get any complication during malaria treatment or had been hospitalized?
7. Yes B. No
8. Do you have any chronic health problems? A. Yes B. No
9. If yes for question no. 6, mention-------------------------
10. For only female participants, are you pregnant? A. Yes B. No
11. Currently, do you have sign and symptoms of malaria disease? A. Yes B. No
12. Which sign and symptoms do you have?
13. Headache B. Fatigue C. Muscle and joint pain D. Shaking and chills

E. Perspiration (sweeting) F. Anorexia (vomiting)

1. Malaria status of the participants? A. positive B. Negative C. Unknown
2. If positive for question no. 9, which species of plasmodium? A. *P. falciparum* B. *P. vivax* C. *P. ovalae* D. *P. malaria* E. mixed (*P.falciparum*/*vivax*)

## Questionnaire (Amharic version)

የተሳታፊ መጠየቆች

ይህ መጠይቅ በአዲስ አበባ ዩኒቨርሲቲ ጤና ሳይንስ ኮሌጅ የህክምና ሳይንስ ት/ቤት የማይክሮባዮሎጂ፤ ኢሚዩኖሎጂ እና ፓራሳይቶሎጂ ትምህርት ክፍል በሜዲካል ፓራሳይቶሎጂ የድህረ-ምረቃ ተማሪ የተዘጋጀ ነዉ፡፡

በቅድሚያ በዚህ ጥናት ላይ ለመሳተፍ ፈቃደኛ በመሆንዎ ላቅ ያለ ምስጋናዬን እያቀረብኩ ከዚህ በመቀጠል ለጥናቱ አስፈላጊ የሆኑ አጠቃላይ እርስዎን የሚገልጹ የስነ-ህዝብ እና የጤና ሁኔታ መረጃዎችን የሚያሳዩ ቃለ መጠይቆች ስላሉኝ በጥንቃቄ በመሙላት እንዲተባበሩኝ እጠይቃለሁ፡፡ እርስዎ የሚሰጡት መረጃ ለጥናቱ ወሳኝ በመሆኑ ትክክለኛዉን መረጃ በጥንቃቄ እንዲሰጡ እየጠየቅሁ እርስዎ የሚሰጡት ማንኛዉም ዓይነት መረጃ ሚስጥራዊነቱ የተጠበቀ እንደሚሆን ላረጋግጥልዎ እወዳለሁ፡፡

የስነ- ህዝብ መረጃዎች እና የጤና ሁኔታ መረጃዎች

- - - 1. የሚስጥር ቁጥር --------------- ዕድሜ…………… ፆታ: ወንድ ሴት
      2. አድራሻ: ከተማ ገጠር
      3. ሄሞግሎቢን ---------------, ግ6ፎዲ---------------------, ግ6ፎዲ/ሄሞግሎቢን ንፅፅር------------------
      4. በወባ በሽታ ታመህ ታዉቃለህ?

ሀ. አዎ ለ. አልታመምኩም

- - - 1. ለጥያቄ ቁጥር 4 መልስዎ አዎ ከሆነ፤ ሆስፒታል ዉስጥ ተኝቶ ለመታከም የሚያበቃ የከፋ ችግር ደርሶብዎት ነበር?

ሀ. አዎ ለ. አልነበረም

- - - 1. ለረጅም ጊዜ የቆየ የጤና ችግር አለብዎት? ሀ. አዎ ለ. የለም
      2. ለጥያቄ ቁጥር 6 መልስዎ አዎ ከሆነ፤ የጤና ችግሩን ይግለፁ-------------------------
      3. አሁን የወባ በሽታ ምልክቶች እና ስሜት አለዎት ? ሀ. አዎ ለ. የለኝም
      4. ተሳታፊዉ አሁን ያለበት የወባ ምርመራ ዉጤት ምንድነዉ? ሀ. በወባ በሽታ ተይዟል ለ.በወባ በሽታ አልተያዘም ሐ. አይታወቅም
      5. ለጥያቄ ቁጥር 9 መልስዎ በወባ በሽታ ተይዟል ከሆነ፤ በየትኛዉ የወባ ዝርያ ነዉ የተያዘዉ?

ሀ. *ፕላስሞዲየምፋልሲፓረም* ለ. ፕላስሞዲየምቫይቫክስ ሐ. *ፕላስሞዲየምኦቫሌ* ሠ. ፕላስሞዲየምማላሬ ረ. ድብልቅ(ፕ.ፋልሲፓረም እና ፕ.ቫይቫክስ)
