# Supplementary material for: Prevalence of G6PD deficiency and distribution of its genetic variants among malaria-suspected patients visiting Metehara health centre, Eastern Ethiopia
Source: Malar J. 2022 Sep 8;21:260. doi: 10.1186/s12936-022-04269-5 (PMC9461287; doi:10.1186/s12936-022-04269-5)
Supplement: Supplementary file 4 — Additional file 4: Laboratory procedures. [file 12936_2022_4269_MOESM4_ESM.docx]

## Procedures for laboratory investigations of G6PD

1. **Procedure for careSTART™ POCT S1 using capillary blood to measure G6PD activity**
   1. Specimen collection
      1. Wash hands with warm water and soap, then dry them completely. Rubbing fingers helps blood being discharged easily;
      2. Follow the lancet’s instructions for use;
      3. Hold the lancet against the fingertip. Press the release button. When you hear a click, the puncture is complete.
      4. Discard a used lancet into safety box.
   2. Measure G6PD/Hb Ratio or G6PD activity

1.2.1. Select
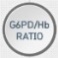
button for starting measurement on the main menu screen;

- - 1. Enter patient identification number using the keypad or barcode scanner and press
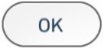
button;


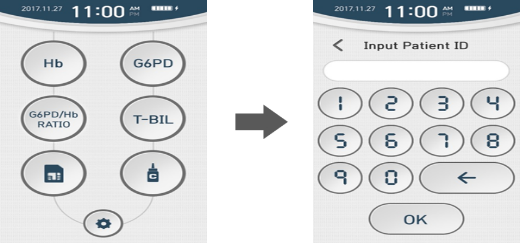


- - 1. Scan G6PD and Hb QR code printed on the test strip vial. If the QR code is read successfully, the code number will be displayed on the screen;


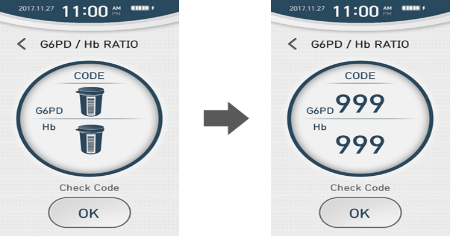


- - 1. Make sure that the code number displayed on the screen matches the code number printed on the test strip vial, then press
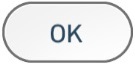
to proceed to the next step;
    2. Take a new careSTART™ POCT S1 G6PD strip and Hb strip from the vial and close the lid after taking out the strip;
    3. Insert the G6PD test strip into the G6PD test strip port, with the ‘G6PD’ label facing upwards. Gently push the test strip into the port until it stops. Then insert the Hb test strip into the Hb test strip port. If the strips are inserted normally, the buzzer will sound and ‘Insert Sample’ screen will be displayed.


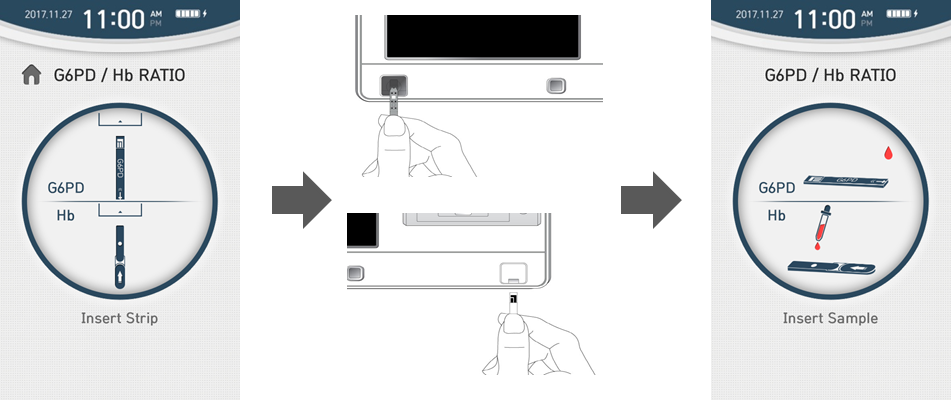

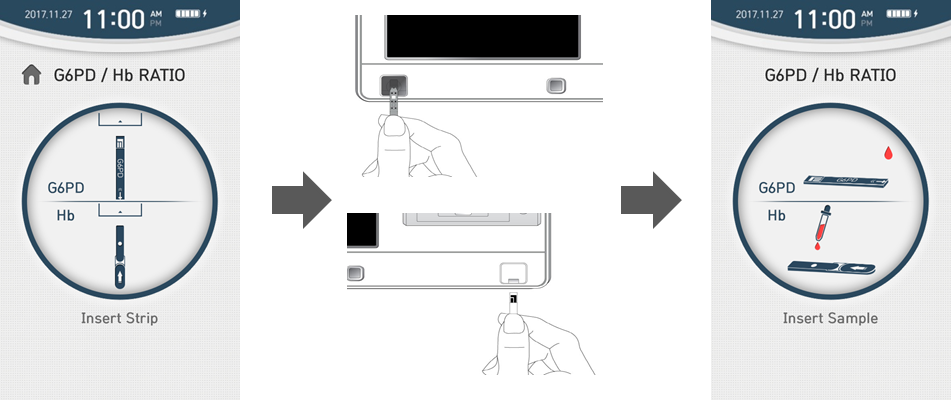


- - 1. Obtain a blood sample using the lancing device. Apply the blood sample into strips. If the sample is loaded successfully, the measurement will begin with a beep sound and the progress bar will be displayed as shown below. When the progress bar reaches 100%, the measurement is completed.


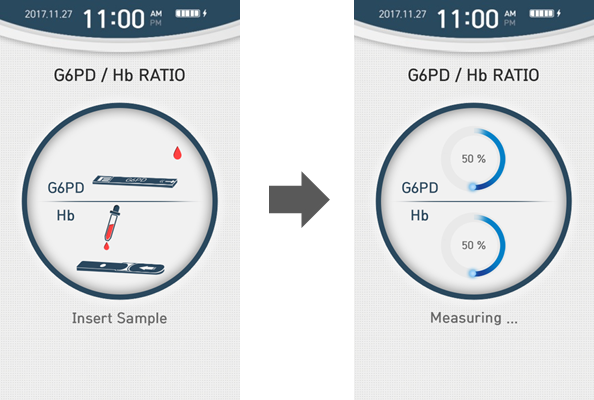


- - 1. When the measurement is completed, the result is displayed on the screen and stored to the memory.


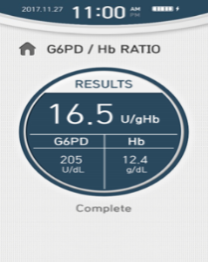


- - 1. Press
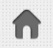
 button to return to the main screen.

1. **Procedure for Genotyping of G6PD variants by molecular method**

PCR reactions were performed to amplify exons 4-11 of the *G6PD* gene to characterize the genotypic mutations.

- 1. DNA was extracted from DBS samples using the PureLink™ Genomic DNA Mini Kit according to manufacturer’s recommendations (Invitrogen ™).
  2. All PCR reactions were performed to a final volume of 20 μl, using 10 μl of 2xMaxima SYBR green PCR Master Mix according to manufacturer’s recommendations, 2 μl of DNA and specific primers for each region.
  3. The PCR conditions used to amplify the three fragments are: 94 °C/3 min, 38 cycles of 94 °C/30 s, 55-58 °C/30 s, and 72 °C/60 s and final extension of 72 °C/6 min.
  4. Amplified PCR products were separated by gel electrophoresis with 1.5% agarose gel at 120 V for 2.00 hours.
  5. Amplified PCR products were purified and sequenced on an ABI 3730xl DNA analyzer based on using published protocols.
  6. Sequences were analyzed BioEdit. All sequences were aligned to the NCBI reference sequence (NG_009015.2) to verify the specificity of the PCR products.
  7. Samples with poor sequencing quality or showed singleton mutations were re-amplified and sequenced. Frequency of all detected mutations will be compared between G6PD normal and deficient patient samples.

## Procedures for malaria diagnostic methods

- - - 1. **Procedure for malaria smear microscopy**

For malaria microscopy laboratory technique thick and thin blood films can be used. Thick blood films are more sensitive in detecting malaria parasites because the blood is concentrated, allowing a greater volume of blood to be examined. Thin blood film is good for species identification of plasmodium species.

- 1. Select clean or new slides, label them with lead pencil on the frosted side of the slide.
  2. Select appropriate site (soft finger or heel for infants) for puncture, clean with 70% alcohol. Puncture, wipe away the first drop with clean gauze.
  3. Collect the second drop by touching it with the side of the slides. Make thick and thin blood films either separately or on the same slide.
  4. Thick and thin blood films were done correctly according to the standard procedure.
  5. Smears were air dried and thin smears were fixed with methanol alcohol for 30 seconds.
  6. Then thick and thin smears were stained with 10% geimsa reagent for 10 minutes and wash with clean water, clean the underside of the slides with gauze, then dry stained slides with air.
  7. The stained smears were investigated with a light microscope by high power magnification (100x) objective to detect the presence of malaria parasites. Thick film preparations were examined first by high power magnification (100x) objective for the presence of parasites.
  8. The results reported with appropriate grading.

1. **Procedure for malaria diagnosis using RDT method**
   1. Make a gentle finger prick of the disinfected site of the not calloused finger with a sterile lancet. Discard the used lancet to appropriate container and by applying gentle pressure remove the first drop of blood.
   2. Using the blood collection device (pipette, inverted cup or capillary tube) Collect 5µl of blood. After pricking and collecting blood, apply a dry cotton wool at the puncture site to stop the bleeding. Discard the blood collection device in the box for infectious waste.
   3. Apply the collected capillary whole blood specimen to the specimen well (circle) of the test device.
   4. Hold the buffer bottle at 90 ̊ (perpendicular) to the test device and not touching the specimen well to avoid contamination. Add 3 drops (90μl) of the buffer into the buffer well (square) of the test device.
   5. Adjust the timer 15 minutes and read the results between 15-30 minutes. Do not read the result after 30 minutes.
   6. Result interpretation
2. The presence of only one colored band ("C" Control line) within the result window indicates a negative result.
3. The presence of two colored bands ("C" Control line and "P.f" *Plasmodium falciparum* line) within the result window, no matter which band appears first, indicates a P.f. positive result.
4. The presence of two colored bands ("C" Control line and "P.v" *Plasmodium vivax* line) within the result window, no matter which band appears first, indicates a P.v positive result.
5. The presence of three colored bands ("C" Control line, “P.f” *Plasmodium falciparum* line and "P.v" *Plasmodium vivax* line) within the result window, no matter which band appears first, indicates a mixed P.f. and P. v positive result.
6. If the control band ("C" Control line) is not visible within the result window, the result is considered invalid. The directions may not have been followed correctly or the test device may have deteriorated. Re-test with a new specimen and a new test device.
